# Sigh maneuver to enhance assessment of fluid responsiveness during pressure support ventilation

**DOI:** 10.1186/s13054-018-2294-4

**Published:** 2019-01-28

**Authors:** Antonio Messina, Davide Colombo, Federico Lorenzo Barra, Gianmaria Cammarota, Giacomo De Mattei, Federico Longhini, Stefano Romagnoli, Francesco DellaCorte, Daniel De Backer, Maurizio Cecconi, Paolo Navalesi

**Affiliations:** 1 Department of Anesthesia and Intensive Care Medicine, IRCCS Humanitas, Humanitas University, Via Alessandro Manzoni, 56, Rozzano, 20089 Milan, Italy; 2 Anesthesia and Intensive Care Medicine, Maggiore della Carità University Hospital, Novara, Italy; 3 Anesthesia and Intensive Care, Azienda Sanitaria Universitaria Integrata, Udine, Italy; 4 Anesthesia and Intensive Care Medicine, Department of Medical and Surgical Sciences, Magna Graecia University, Catanzaro, Italy; 5 Department of Anesthesia and Intensive Care, University of Florence, Azienda Ospedaliero-Universitaria Careggi, Florence, Italy; 6 Intensive Care Departments CHIREC Hospitals, Brussels, Belgium

**Keywords:** Fluid responsiveness, Functional hemodynamic assessment, Sigh maneuver, Pressure support ventilation

## Abstract

**Background:**

Assessment of fluid responsiveness is problematic in intensive care unit (ICU) patients, in particular for those undergoing modes of partial support, such as pressure support ventilation (PSV). We propose a new test, based on application of a ventilator-generated sigh, to predict fluid responsiveness in ICU patients undergoing PSV.

**Methods:**

This was a prospective bi-centric interventional study conducted in two general ICUs. In 40 critically ill patients with a stable ventilatory PSV pattern and requiring volume expansion (VE), we assessed the variations in arterial systolic pressure (SAP), pulse pressure (PP) and stroke volume index (SVI) consequent to random application of 4-s sighs at three different inspiratory pressures. A radial arterial signal was directed to the MOSTCARE™ pulse contour hemodynamic monitoring system for hemodynamic measurements. Data obtained during sigh tests were recorded beat by beat, while all the hemodynamic parameters were averaged over 30 s for the remaining period of the study protocol. VE consisted of 500 mL of crystalloids over 10 min. A patient was considered a responder if a VE-induced increase in cardiac index (CI) ≥ 15% was observed.

**Results:**

The slopes for SAP, SVI and PP of were all significantly different between responders and non-responders (*p* < 0.0001, *p* = 0.0004 and *p* < 0.0001, respectively). The AUC of the slope of SAP (0.99; sensitivity 100.0% (79.4–100.0%) and specificity 95.8% (78.8–99.9%) was significantly greater than the AUC for PP (0.91) and SVI (0.83) (*p* = 0.04 and 0.009, respectively). The SAP slope best threshold value of the ROC curve was − 4.4° from baseline. The only parameter found to be independently associated with fluid responsiveness among those included in the logistic regression was the slope for SAP (*p* = 0.009; odds ratio 0.27 (95% confidence interval (CI_95_) 0.10–0.70)). The effects produced by the sigh at 35 cmH_2_0 (Sigh_35_) are significantly different between responders and non-responders. For a 35% reduction in PP from baseline, the AUC was 0.91 (CI_95_ 0.82–0.99), with sensitivity 75.0% and specificity 91.6%.

**Conclusions:**

In a selected ICU population undergoing PSV, analysis of the slope for SAP after the application of three successive sighs and the nadir of PP after Sigh_35_ reliably predict fluid responsiveness.

**Trial registration:**

Australian New Zealand Clinical Trials Registry, ACTRN12615001232527. Registered on 10 November 2015.

**Electronic supplementary material:**

The online version of this article (10.1186/s13054-018-2294-4) contains supplementary material, which is available to authorized users.

## Background

Assessing preload dependence in critically ill patients is a challenge for intensive care unit (ICU) physicians [1, 2]. During controlled mechanical ventilation, dynamic indexes can be applied in non-arrhythmic patients with sufficiently high tidal volume (V_T_), i.e., > 8 mL/kg body weight and non-severely impaired lung compliance [3–8]. The interplay between mechanical ventilation and hemodynamics is more complex in patients with spontaneous breathing activity, whose respiratory efforts affect intrathoracic pressure and venous return to the right ventricle (RV) [9–12]. To overcome these limitations, functional hemodynamic assessment, consisting of maneuvers determining a sudden change in cardiac preload, such as passive leg raising (PLR) or end-expiratory occlusion test (EEOT), represents a valuable means of assessment of fluid responsiveness [13–15].

Both PLR and EEOT have been successfully utilized for assessing fluid responsiveness, regardless of ventilatory assistance and mode of ventilation [15, 16]. Unfortunately, however, some drawbacks limit the extensive use of these maneuvers in clinical practice. One the one hand, PLR cannot be applied in some clinical situations, such as trauma of the hip, legs or lumbar spine, deep venous thrombosis and intracranial or abdominal hypertension [17–20]. Indeed, a recent large observational study showed PLR to be the most common form of assessment of fluid responsiveness, being used, nonetheless, in only 10.7% of the patients needing the assessment of fluid responsiveness [2]. On the other hand, rates of EEOT failure as high as 22.5% have been reported, consequent to visible patient’s effort against the occluded airway [15].

We propose here a new approach for assessing fluid responsiveness in patients undergoing partial ventilatory assistance. We hypothesized that the changes from baseline in systolic arterial pressure (SAP), pulse pressure (PP) and stroke volume index (SVI) in relationship to the airway pressure (Paw) generated during a “sigh” maneuver [21] can predict fluid responsiveness in ICU patients undergoing pressure support ventilation (PSV).

## Materials and methods

### Setting and design

The study was performed in the ICUs of two Italian University Hospitals (AOU Maggiore della Carità of Novara and AOU Careggi of Firenze) after approval of the institutional ethics committees (protocol numbers 149/14 and 2014/0035819 for Novara and Firenze, respectively), in accordance with the principles of the Declaration of Helsinki. The patient’s written consent was managed as indicated by the ethics committees. AM and SR enrolled the patients.

Patients were included when they met all the following criteria: (1) mechanical ventilation ≥ 48 h; (2) indication for volume expansion (VE) according to the attending physician’s decision, based on the presence at least one of the following signs of inadequate tissue perfusion: (a) systolic blood pressure < 90 mmHg (or a decrease > 50 mmHg from baseline in hypertensive patients, (b) need for dopamine > 5 mcg/kg/min or norepinephrine at any dosage; (c) urine output < 0.5 mL/kg/h for ≥ 2 h; (d) tachycardia > 100/ min or (e) presence of skin mottling [14, 15, 22] and (3) PSV with inspiratory support level (PS) between 8 and 15 cmH_2_O and positive end-expiratory pressure (PEEP) between 5 and 10 cmH_2_O. Exclusion criteria were (1) severe myocardial or valvular dysfunction; (2) cardiac arrhythmias; (3) severe acute respiratory distress syndrome (ARDS); (4) hemodialysis or continuous hemofiltration; (5) body mass index ≥ 30 and (6) altered arterial signal recording (patients excluded from data analysis).

### Study protocol and measurements

In all patients, PSV was applied using the Maquet Servo-I ventilator (Maquet Critical Care, Solna, Sweden), which displays online airway pressure, flow and volume waveforms. The protocol was started during a period of stable ventilatory pattern, defined by a median variation of respiratory rate, tidal volume and minute ventilation < 15% in the hour preceding patient enrolment as assessed by displaying trends on the ventilator screen. Three sighs, in a computer-generated random order, at either 15 (Sigh_15_), 25 (Sigh_25_) and 35 (Sigh_35_) cmH_2_O of total inspiratory Paw (PEEP + PSV) were delivered. To add the sigh to PSV, we set the ventilator pressure-controlled synchronized intermittent mandatory ventilation plus PSV (SIMV (PC) + PS mode), with SIMV rate set a 1/min and inspiratory time of 4 s, as previously described [21]. The sigh was manually marked on MOSTCARE™ and the ventilator contemporaneously switched from PSV to SIMV(PC) + PSV, by two different investigators.

After the end of the last sigh maneuver, VE was performed using 500 mL of crystalloids over 10 min. The test was repeated if cough, visible respiratory efforts or cardiac arrhythmias occurred during the maneuver. Persistent cough or cardiac arrhythmias determined the patient’s withdrawal.

The arterial waveform signal was obtained from a 20-gauge cannula inserted in the radial artery (Leadercath Arterial polyethylene catheter 20 gauge, 8 cm length, 0.6 mm internal diameter × 0.9 mm external diameter; Vygon, Ecouen, France) and connected to a disposable pressure transducer (Package transducer Edwards; VAMP Plus system; Edwards Lifesciences, Irvine, California). The signal was directed to the MOSTCARE™ pulse contour hemodynamic monitoring system (Vygon, Vytech Health, Padova, Italy) to assess SAP, PP and SVI variations. The MOSTCARE™ analyzes the arterial pressure wave to identify the “points of instability” distributed along the wave profile. These points represent the interaction between forward waves (due to cardiac systole) and backward waves coming from the periphery. Analysis of the waveform profile estimates the vascular impedance and the hemodynamic parameters. The MOSTCARE™ algorithm calculates SVI using a beat-by-beat analysis of the systolic portion of the arterial waveform at a sampling rate of 1000 Hz. MOSTCARE™ extracts all arterial pressures directly from the pressure waveform while it calculates PP variation [23, 24].

A square-wave test was performed in all patients before starting the study protocol to avoid the risk of under-damping or over-damping of the arterial pressure transducer and the consequent inaccurate hemodynamic assessment [25]. Data were downloaded using dedicated software (MOSTCARE™ Data Card Reader® 4.0.11). The acquisition software was set to record beat by beat during the sigh tests only, while it averaged all the hemodynamic parameters over 30 s for the remaining period of the study protocol.

### Statistical analysis

Data are expressed as median and 25th–75th interquartile range, unless otherwise specified. Data were compared between responders and non-responders using the Mann–Whitney or Wilcoxon’s rank sum test. The chi-square test for comparison of proportions was applied for analysis of dichotomous or categorical variables.

To test our hypothesis, we predicted an area under the receiver operating characteristic (ROC) curve (AUC) for Sigh_35_ of at least 0.75, which is the threshold for considering a diagnostic test as accurate [26]. To calculate the sample size of the study, we compared this value to the null hypothesis (AUC = 0.50; meaning no discriminating power; ratio of sample sizes in negative/positive groups = 1). A sample of 38 patients was determined to be necessary to address our study aim (type I error of 5% and type II error of 20%).

**Fig. 1:**
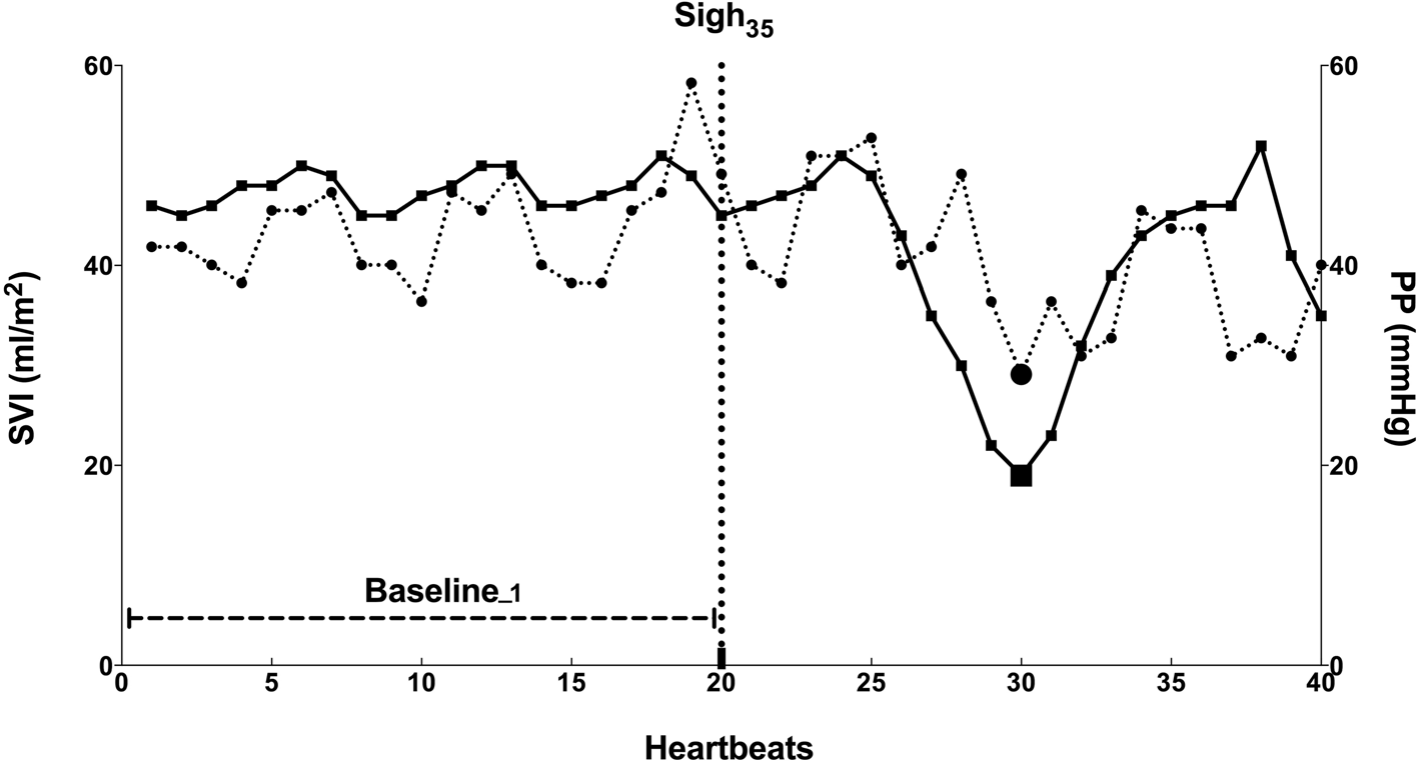
Schematic illustration of pulse pressure (PP) and stroke volume index (SVI) variations after Sigh_35_ in one responder. The solid line with squares indicates PP and the dotted line with circles depicts SVI. Baseline_1 corresponds to the 20 heartbeats preceeding Sigh_35_, which starts at heartbeat number 20, as indicated by the dotted vertical line. Nadir values of PP and SVI are indicated by the larger square and circle, respectively. Both PP and SVI dropped after application of Sigh_35_

The hemodynamic effect of each sigh on SAP, SVI and PP was evaluated considering the mean of the 20 beats before each sigh (baseline_15_, baseline_25_ and baseline_35_ for Sigh_15_, Sigh_25_ and 35 Sigh_35_, respectively) and the nadir value of the 20 beats after sigh application (Fig. 1).

**Fig. 2:**
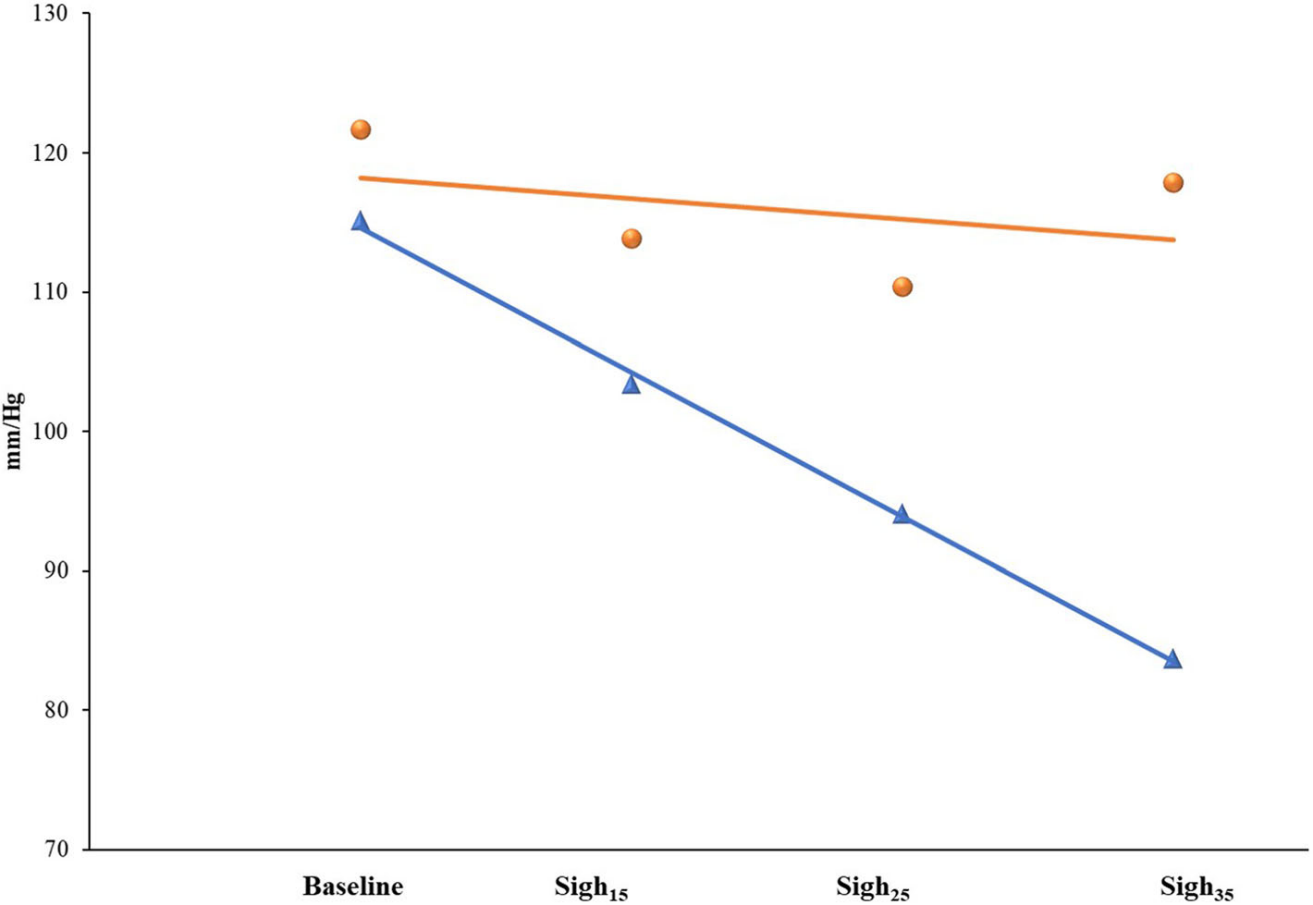
Slope calculation of responders (blue line) and non-responders (red line) of systolic arterial pressure (SAP). The blue triangles and the red circles represent the mean values of the two populations (responders and non-responders, respectively) at each step of the protocol. The AUC for the slope of SAP (0.99; sensitivity 100.0% (79.4–100.0%) and specificity 95.8% (78.8–99.9%)) was significantly greater than the AUCs for PP (0.91) and SVI (0.83) (*p* = 0.04 and 0.009, respectively). The SAP slope best threshold value of the ROC curve was − 4.4° from baseline

The slope of SAP, SVI and PP (angle in degrees) was calculated by plotting the nadir value of each variable against the corresponding airway pressure (15, 25 and 35 cmH_2_0 for Sigh_15_, Sigh_25_ and 35 Sigh_35_, respectively) and considering the baseline of the first sigh delivered to each patient as the starting point (Fig. 2 and Additional file 1: Figures S1 and S2).

ROC curves (CI_95_) were constructed based on (1) the percent changes in SAP, PP, and SVI between baseline and nadir and (2) the slope for each patient versus the response to VE administration. A patient was considered a responder if a VE-induced increase in CI ≥ 15% was observed [5]. ROC curves were compared using the Hanley–McNeil test. The three baselines (baseline_15_, baseline_25_ and baseline_35_) were compared by means of analysis of variance (ANOVA) for repeated measures. Logistic regression analysis with a backward stepwise approach was used to identify the association between the nadirs and the slopes of SAP, PP and SVI and the fluid responsiveness.

Statistical analyses were conducted using GraphPad PRISM V6 (GraphPad Software Inc., San Diego, CA, USA) and Medcalc (Software 8.1.1.0; Mariakerke, Belgium).

## Results

Forty-five adult patients were enrolled; however, only 40 patients were included in the data analysis. In fact, we excluded two patients because of arrhythmia after enrolment, and three because they had a persistent cough after sigh application. No patient was excluded from data analysis because of distorted arterial waveform signal.

Table 1 displays patients’ characteristics at enrolment, while Additional file 2: Table S1 in the supplementary material displays the hemodynamic measurements at each step of the protocol. A total of 27 patients (10 responders and 17 non-responders) did not receive any vasoactive drug. The median Richmond Agitation Sedation Scale (RASS) score was − 2 ± 0.5 in responders and − 2 ± 0.6 in non-responders (*p* = 0.27). The hemodynamic effects of VE in responders and non-responders are separately reported in Table 2. The AUC for PP variation during the period of stable ventilatory pattern before sigh application was 0.51 (CI_95_ 0.34–0.67). The nadirs of SAP, SVI and PP occurred within the first 10 of the 20 beats analyzed after Sigh_25_ in 15 of 16 (93.7%) responders and 19 of 24 (79.1%) non-responders (*p* = 0.22), while after Sigh_35_ in 13 of 16 (81.2%) responders and 17 of 24 (70.8%) non-responders (*p* = 0.47). SAP, SVI and PP values at baseline_15_, baseline_25_ and baseline_35_ did not differ (*p* = 0.27, 0.28 and 0.12, respectively).

**Table 1:** Patient characteristics at enrolment

General characteristics	Responders (*n* = 16)	Non-responders (*n* = 24)	*p* value
Age (years)	72 [70–77]	64 [54–75]	0.34
Gender (male/female)	14/2	14/10	0.07
Body mass index (kg/m^2^)	24 [21–27]	24 [23–26]	0.85
SAPS II	46 [44–49]	45 [41–48]	0.43
Temperature (°C)	37.2 [36.8–37.4]	36.9 [36.1–37.3]	0.45
Ventilator settings (baseline)			
PEEP (cmH_2_O)	5.0 [5.0–7.0]	5.0 [5.0–6.3]	0.77
Pressure support (cmH_2_O)	10.0 [8.8–10.0]	10.0 [10.0–10.0]	0.82
V_T_ (mL/kg ideal body weight)	6.8 [5.9–7.4]	7.0 [6.6–7.9]	0.34
PaO_2_^/^FiO_2_ (ratio)	310 [280–354]	302 [274–330]	0.65
RR (breaths/min)	15 [13–18]	13 [10–15]	0.02
HR/RR ratio	6.2 [5.0–6.5]	5.9 [5.0–6.4]	0.71
Vasoactive agents, *n*; (μg kg^− 1^ min^− 1^)			
Norepinephrine	6; (0.2 [0.2–0.3])	7; (0.2 [0.1–0.2])	0.85
Dopamine	2; (5.6 [4.8–5.9])	0	0.89
Acute circulatory failure origin, *n*; (%)			
Sepsis/septic shock	6 (37.5)	10 (41.7)	0.99
Hypovolemia	7 (43.8)	8 (33.3)	0.53
Trauma	0	1 (4.1)	0.99
Intracranial diseases	3 (18.7)	5 (20.9)	0.99

Data presented as median (25th–75th IQR) unless otherwise specified

*IQR* interquartile, *SAPS* simplified acute physiology score, *PEEP* positive end-expiratory pressure, *V*_*T*_ tidal volume, *PaO*_*2*_^*/*^*FiO*_*2*_ arterial partial pressure of oxygen/fraction of inspired oxygen, *RR* respiratory rate, *HR* heart rate

**Table 2:** Effects of fluid administration on hemodynamic parameters in fluid responders and non-responders

Hemodynamic variables	Pre VE	Post VE	*p* values R and NR baseline	*p* values Pre and postVE
CI (L/min/m^2^)				
Responders	2.1 [2.0–2.2]	2.8 [2.5–3.4]	0.0006	0.0005
Non-responders	2.8 [2.4–2.9]	2.7 [2.4–2.9]		0.24
SVI (mL/m^2^)				
Responders	24 [21–26]	38 [29–41]	0.001	0.0006
Non-responders	34 [28–40]	35 [28–41]		0.08
MAP (mmHg)				
Responders	71 [64–76]	88 [80–94]	0.13	0.0005
Non-responders	77 [69–86]	81 [72–89]		0.003
SAP (mmHg)				
Responders	106 [98–124]	132 [122–149]	0.23	< 0.0001
Non-responders	115 [106–126]	128 [113–140]		< 0.0001
HR (beats/min)				
Responders	90 [83–92]	85 [79–88]	0.01	0.02
Non-responders	73 [63–87]	71 [64–82]		0.14
PPV (%)				
Responders	8.2 [5.6–14.8]	7.0 [5.8–10.6]	0.94	0.04
Non-responders	9.8 [7.0–17.4]	5.1 [4.3–7.7]		0.007

Data presented as median (25th – 75th IQR) unless otherwise specified

*IQR* interquartile, *R* responders, *NR* non-responders, *VE* volume expansion, *CI* cardiac index, *SVI* stroke volume index, *MAP* mean arterial pressure, *SAP* systolic arterial pressure, *HR* heart rate, *PPV* pulse pressure variation, *VE* volume expansion

### Effects of sigh application (see Table 3)

The variations of SAP, SVI and PP changes after Sigh_15_ were not different between responders and non-responders and, therefore, the ROC curves were not calculated.

**Table 3:** Variations in SAP, PP and SVI and slope following sigh application in responders and non-responders

Hemodynamic variables	Responders	Non-responders	*p* value	AUC (CI_95_)	ROC-curve sensitivity (%)	ROC-curve specificity (%)	ROC-curve best cutoff
Sigh_15_							
Nadir of SAP (%)	−5.8 (− 12.3/−3.2)	−4.8 (−6.5/−2.5)	0.19	NA	NA	NA	NA
Nadir of PP (%)	−10.7 (− 18.6/−6.0)	−6.3 (− 14.3/−4.1)	0.10	NA	NA	NA	NA
Nadir of SVI (%)	−1.7 (−2.6/−0.9)	− 1.9 (−2.5/−1.0)	0.91	NA	NA	NA	NA
Sigh_25_							
Nadir of SAP (%)	− 18.0 (−8.6/− 21.8)	−13.8 (−7.3/−18.3)	0.004	0.77 (0.61–0.93)	50.0 (29.1–70.8)	87.5 (61.5–98.4)	−17%
Nadir of PP (%)	−23.8 (−15.3/−38.6)	−23.4 (− 12.3/−26.3)	0.002	0.78 (0.63–0.93)	70.8 (48.9–87.0)	75.0 (47.6–92.0)	− 17%
Nadir of SVI (%)	−1.0 (−9.8/8.2)	−8.7 (−3.2/−15.8)	0.38	0.58 (0.40–0.77)	NA	NA	NA
Sigh_35_							
Nadir of SAP (%)	− 24.9 (−19.3/−31.0)	−13.8 (− 7.3/−18.3)	0.0003	0.83 (0.70–0.95)	62.5 (35.4–84.0)	91.6 (73.0–98.0)	− 14%
Nadir of PP (%)	−38.9 (− 35.1/−53.5)	−23.4 (− 12.3 / -26.3)	< 0.0001	0.91 (0.82–0.99)	75 (47.6–92.7)	91.6 (73.0–98.9)	−35%
Nadir of SVI (%)	−22.8 (− 13.7/−27.5)	−8.7 (− 3.2/−15.8)	0.0002	0.83 (0.71–0.95)	68.7 (41.3–88.9)	87.5 (67.4–97.0)	−21%
Slope							
Slope of SAP	−10.4° (− 11.9/−8.6)	−1.5° (− 3.0/−0.20)	< 0.0001	0.99 (0.99–1.01)	100.0 (79.4–100.0)	95.8 (78.8–99.9)	−4.4°
Slope of PP	− 7.6° (− 9.3/−6.4)	−3.7° (− 5.1/−2.1)	< 0.0001	0.91 (0.77–0.97)	68.7 (41.3–88.9)	95.8 (78.8–99.9)	−7.0°
Slope of SVI	−3.3° (− 3.8/−2.2)	−1.5° (− 2.5/−0.9)	< 0.0001	0.83 (0.68–0.93)	56.2 (29.8–80.2)	91.6 (73.0–98.9)	−3.2°

Data presented as median (25th–75th IQR) and as percentage of variation with respect to baseline (nadir, see text for further explanations). *SAP* systolic arterial pressure, *PP* pulse pressure, *SVI* stroke volume index, *AUC* area under the curve, *CI* confidence interval, *ROC* receiver operating characteristic, *NA* not applicable

After Sigh_25_, the reductions in SAP and PP were statistically significant between responders and non-responders [(− 18.0% (− 8.6/− 21.8) vs*.* − 13.8% (− 7.3/− 18.3); *p* = 0.004 and − 23.8% (− 15.3/− 38.6) vs. − 23.4% (− 12.3/− 26.3); *p* = 0.002, respectively]), while the reduction in SVI was not (*p* = 0.38). After Sigh_35_, reductions in SAP, SVI and PP were all significantly different between responders and non-responders [(24.9% (− 19.3/− 31.0) vs. − 13.8% (− 7.3/− 18.3); *p* = 0.0003, − 22.8% (− 13.7/− 27.5) vs. − 8.7% (− 3.2/− 15.8); *p* = 0.0002 and − 38.9% (− 35.1/− 53.5) vs. − 23.4% (− 12.3/− 26.3); *p* < 0.0001, respectively)]. The AUCs of the ROC obtained after Sigh_25_ were not significantly different for any of the variables analyzed. The AUC for PP after Sigh_35_ [(0.91 (0.82–0.99); sensitivity 75% (47.6–92.7%) and specificity 91.6 (73.0–98.9%)] was significantly greater than the AUCs for SAP [(0.83 (0.70–0.95) and SVI 0.83 (0.71–0.95); *p* = 0.03 for both the comparisons)]. The PP nadir best threshold value of the ROC curve was − 35% from baseline.

The slopes of SAP, SVI and PP were all significantly different between responders and non-responders [(− 10.4°(− 11.9/− 8.6) vs. − 1.5°(− 3.0/− 0.20); − 3.3°(− 3.8/− 2.2) vs. − 1.5°(− 2.5/− 0.9) and 7.6° (− 9.3/− 6.4) vs. − 3.7°(− 5.1/− 2.1), respectively; *p* < 0.0001 for all comparisons)]. The AUC of the slope for SAP [(0.99 (0.99–1.01); sensitivity 100.0% (79.4–100.0%) and specificity 95.8% (78.8–99.9%)] was significantly greater than the AUCs for PP [(0.91 (0.77–0.97)) and SVI (0.83 (0.68–0.93); *p* = 0.04 and 0.009, respectively)]. The SAP slope best threshold value of the ROC curve was − 4.4° from baseline (Fig. 2).

The only parameter found to be independently associated with fluid responsiveness among those included in the logistic regression was the slope for SAP [(*p* = 0.009; odds ratio 0.27 (CI_95_ 0.10–0.70)].

## Discussion

Our study shows that the functional hemodynamic assessment of (1) the lowest SAP values obtained after the consecutive application of sighs at 15, 25 and 35 cmH_2_O and of (2) the lowest PP valued obtained after one sigh at and 35 cmH_2_O reliably predict fluid responsiveness in a selected ICU population undergoing PSV.

The rate of the dynamic indexes of fluid responsiveness being used in ICU patients to assess fluid responsiveness is rather small [8]. Reliable tests are available for patients undergoing controlled mechanical ventilation such as PLR [20], EEOT [15, 27, 28] and, more recently, the “tidal volume challenge” [29] and “mini-fluid challenge” [30].

For the increasing number of ICU patients retaining, to some extent, a spontaneous breathing activity [8, 31, 32], only PLR has been repeatedly demonstrated effective [17–20], while the EEOT reliably predicted fluid responsiveness in those patients able to maintain a 15-s respiratory occlusion without triggering the ventilator [33, 34] or with absence of spontaneous breathing efforts during the maneuver [35]. Spontaneous breathing activity affects the reliability of the dynamic indexes of fluid responsiveness by influencing V_T_ magnitude [7], increasing respiratory rate and, therefore, reducing heart rate/respiratory rate ratio^34^ [36] and causing asynchronies between patient and ventilator^11^ [12]. In fact, in the present study, the AUC was 0.51 for PP variation, which makes this index unsuitable for clinical use, confirming that fluid responsiveness should always be assessed by a functional test in patients with spontaneous breathing activity.

This is the first study demonstrating the feasibility of applying a transient increase in V_T_ by adding a sigh to predict fluid responsiveness in ICU patients with residual spontaneous breathing activity and undergoing PSV. In fact, a similar approach has been already successfully used in patients undergoing forms of controlled ventilation to enhance the reliability of stroke volume variation and PP variation. Freitas et al. increased the V_T_ from 6 to mL to 8 mL/kg body weight for 5 min [37]; Reuter et al. randomly applied 5, 10, and 15 mL/kg of V_T_ [38] and, more recently, Myatra et al. used a 1-min “V_T_-challenge”, increasing the V_T_ from 6 up to 8 mL/kg [39]. Finally, the analysis of the slope for SAP nadir has previously been successfully applied in small cohorts of postsurgical patients [40–42].

In a similar manner, the application of a sigh is a test aimed at revealing fluid responsiveness by inducing increases in V_T_ and in intrathoracic pressure, which reduces the stroke volume by increasing RV afterload and, to some extent, by reducing RV preload. The RV is extremely sensitive to an acute increase in afterload and is unable to maintain the systolic function in this condition and reduces RV stroke volume through a beat-to-beat adaptive response [43]. The complex interplay between sigh application and the transmission of the increased intrathoracic pressure to the RV would also cause false positive results in those patients affected by RV failure, by causing an amplified decrease in RV stroke volume.

After a few heartbeats, the reduced RV stroke volume affects left ventricle preload and SV, and, consequently, SAP and PP. The PP nadir occurred within the first 10 heartbeats after sigh application in 80% of our patients [3, 4]. For instance, the decrease in both PP and SVI after a recruitment maneuver delivered with a positive airway pressure of 30 cm H_2_O for 30 s has been recently successfully applied in patients undergoing general anesthesia, to predict fluid responsiveness [44].

The hemodynamic variations induced by Sigh_15_ on PP and SVI were negligible, while those induced by Sigh_25_, though statistically significant, were small and overall were insufficient for clinical use (Table 3). After Sigh_35,_ the AUCs for SAP and SVI were almost identical, whereas the AUC for PP was larger AUC consequent to a higher specificity (91.6%). The variable transmission of the applied inspiratory pressure may explain the lack of reliability of Sigh_35_ in some patients. Lansdorp et al. demonstrated that the amount of Paw distributed to the pericardium and vena cava is about one third of the overall applied pressure, with ± 17% and ± 11% variability for pericardium and vena cava, respectively [45]. If, on the one hand, Sigh_35_ identifies those patients who should not receive fluids to correct hemodynamic instability, i.e., displaying a drop in PP ≤ 35% during Sigh_35_, on the other hand, it fails to recognize some responders, which makes an adjunctive form of functional hemodynamic assessment such as the PLR advisable before administering fluids.

The analysis of the slope for SAP variations after sigh application is a post-hoc mathematical elaboration, which predicts fluid responsiveness. The evaluation only of the nadir values avoids the inclusion of early inspiratory increases in the stroke volume, which are more prominent in patients with hypervolemia and congestive heart failure and not related to volume responsiveness [11, 46]. The values of the slope angle are generated by the decrease in SAP during elevation of intrathoracic pressure after sigh application and are linked to the position of the ventricle on the Frank–Starling curve (the higher the value, the larger the preload dependence). However, Trepte et al. applied three consecutive pressure-controlled mechanical breaths of gradually increasing pressure up to 30 cmH_2_0, demonstrating moderate reliability of the nadir SAP analysis in predicting fluid responsiveness (AUC 0.77) [42]. Though reliable overall, this analysis of the slope is limited by potential technical limitations. First, bedside clinical application of the slope analysis should be based on automatic computation of SAP changes, which is not presently available for routine use. However, in principle it would be possible to determine the SAP nadir during the first 20 beats after each sigh and obtain the slope measurement in a few minutes by means of a spreadsheet. Second, correct computation is affected by the occurrence of extrasystoles and by the precision of the measurement (the best cutoff value is − 4.4°) during the application of three consecutive pressure-controlled mechanical breaths. However, these technical limitations could be easily overcome by software integrating the signals obtained by the ventilator and the cardiac output monitoring device, making the sigh test easily applicable at the bedside.

Some may argue about the safety of raising Paw to 35 cmH_2_O, though for a few seconds. In 13 ICU patients with early ARDS, Patroniti et al. increased Paw once a minute during PSV for 3–5 s at a minimum of 35 cm H_2_O and observed an improvement in oxygenation without adverse effects [21]. As confirmed, none of our patients had complications or side effects related to sigh application.

Our study has some limitations. First of all, our criteria for patient selection are very strict, as it is commonly the case for “proof of concept” studies, which often include relatively few and highly selected patients in order to control potential confounding factors, limiting the external validity of the study. The inclusion and exclusion criteria have been specifically requested by the local ethical committees to guarantee the safety of the study protocol application. Unfortunately, this led to a selection of less severely ill ICU patients. Second, the reliability of MOSTCARE™ is still debated. Despite the positive results of a large multicenter study performing a head-to-head comparison with transthoracic echocardiography along five consecutive heartbeats [24], the ability of MOSTCARE™ in tracking CI variations during fluid infusion is still questioned [47], Moreover, the MOSTCARE™ reliability is strictly dependent on the quality of the arterial pulse and on the ability of the operator to recognize artifacts of the signal and the centers involved are highly trained in the MOSTCARE™ use. Finally, we assessed the fluid responsiveness by infusing a VE of 500 mL over 10 min, in line with several previous ICU studies adopting the VE as gold standard [48]. However, recently, Aya et al. tested different doses of VE and obtained different proportions of responders and non-responders [49]. Since we did not adjust the VE on the body weight, some patients could be under or over-challenged, potentially affecting the rate of fluid responsiveness and, in turn, the ROC curve analysis.

For all these reasons, the promising results of this pilot investigation need to be confirmed in studies with less selective inclusion criteria.

## Conclusions

The slope of the line defined by SAP and airway pressure after the application of three successive sighs and the nadir of PP after a sigh with 35 mmHg can predict fluid responsiveness in patients mechanically ventilated with spontaneous breathing. Assessments of applicability and effectiveness in larger patient populations and comparisons with other functional tests of fluid responsiveness are deemed necessary to ascertain the clinical importance of these findings.

## Additional file

Additional file 1: **Figures S1** and **S2.** Slope calculation of pulse pressure (PP) and stroke volume index (SVI) in responders and non-responders. Red triangles and green circles represent the mean values of PP the two populations (responders and non-responders, respectively) at each step of the protocol. Purple triangles and yellow circles represent the mean values of SVI the two populations (responders and non-responders, respectively) at each step of the protocol. For slope computations, please refer to Table 3. (ZIP 57 kb)

Additional file 2: **Table S1.** Hemodynamic baseline values at each step of the protocol. (DOCX 15 kb)

## Abbreviations

ARDS: Acute respiratory distress syndrome
AUC: Area under the curve
CI: Cardiac index
CI_95_: 95% confidence interval
EEOT: End-expiratory occlusion test
ICU: Intensive care unit
Paw: Airway pressure
PEEP: Positive end-expiratory pressure
Pes: Esophageal pressure
PLR: Passive leg raising
PP: Pulse pressure
PS: Pressure support
PSV: Pressure support ventilation
RASS: Richmond agitation sedation scale;
ROC: Receiver operating characteristic
RV: Right ventricle
SAP: Systolic arterial pressure
SV: Stroke volume
SVI: Stroke volume index
VE: Volume expansion
V_T_: Tidal volume
