# Supplementary figures and images for: Sigh maneuver to enhance assessment of fluid responsiveness during pressure support ventilation
Source: Crit Care. 2019 Jan 28;23:31. doi: 10.1186/s13054-018-2294-4 (PMC6350369; doi:10.1186/s13054-018-2294-4)

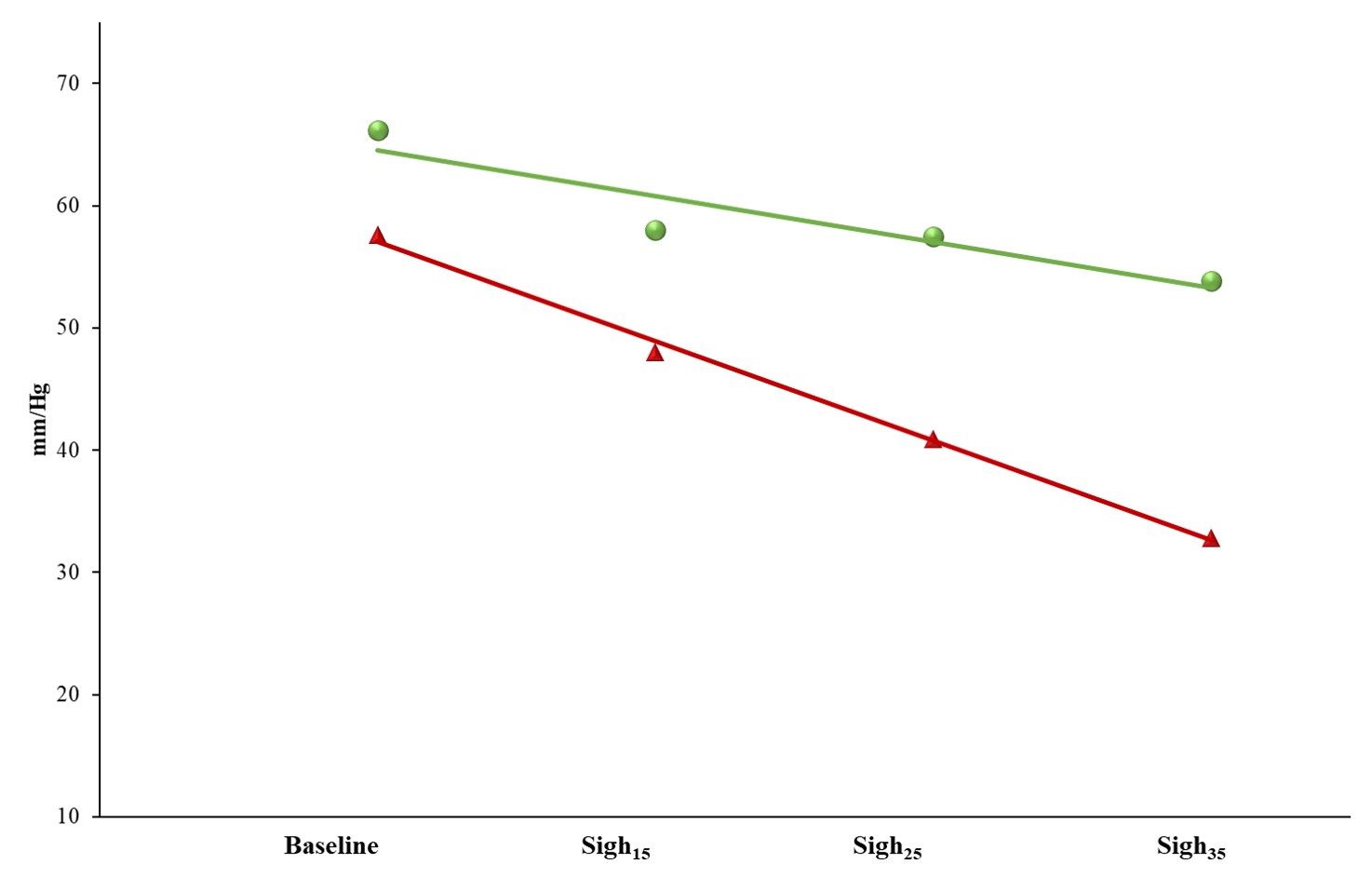

Supplement: Supplementary file 1 — Figures S1 and S2. Slope calculation of pulse pressure (PP) and stroke volume index (SVI) in responders and non-responders. Red triangles and green circles represent the mean values of PP the two populations (responders and non-responders, respectively) at each step of the protocol. Purple triangles and yellow circles represent the mean values of SVI the two populations (responders and non-responders, respectively) at each step of the protocol. For slope computations, please refer to Table 3. (ZIP 57 kb) [file 13054_2018_2294_MOESM1_ESM.zip › Suppl_fig_1_PP.jpg]

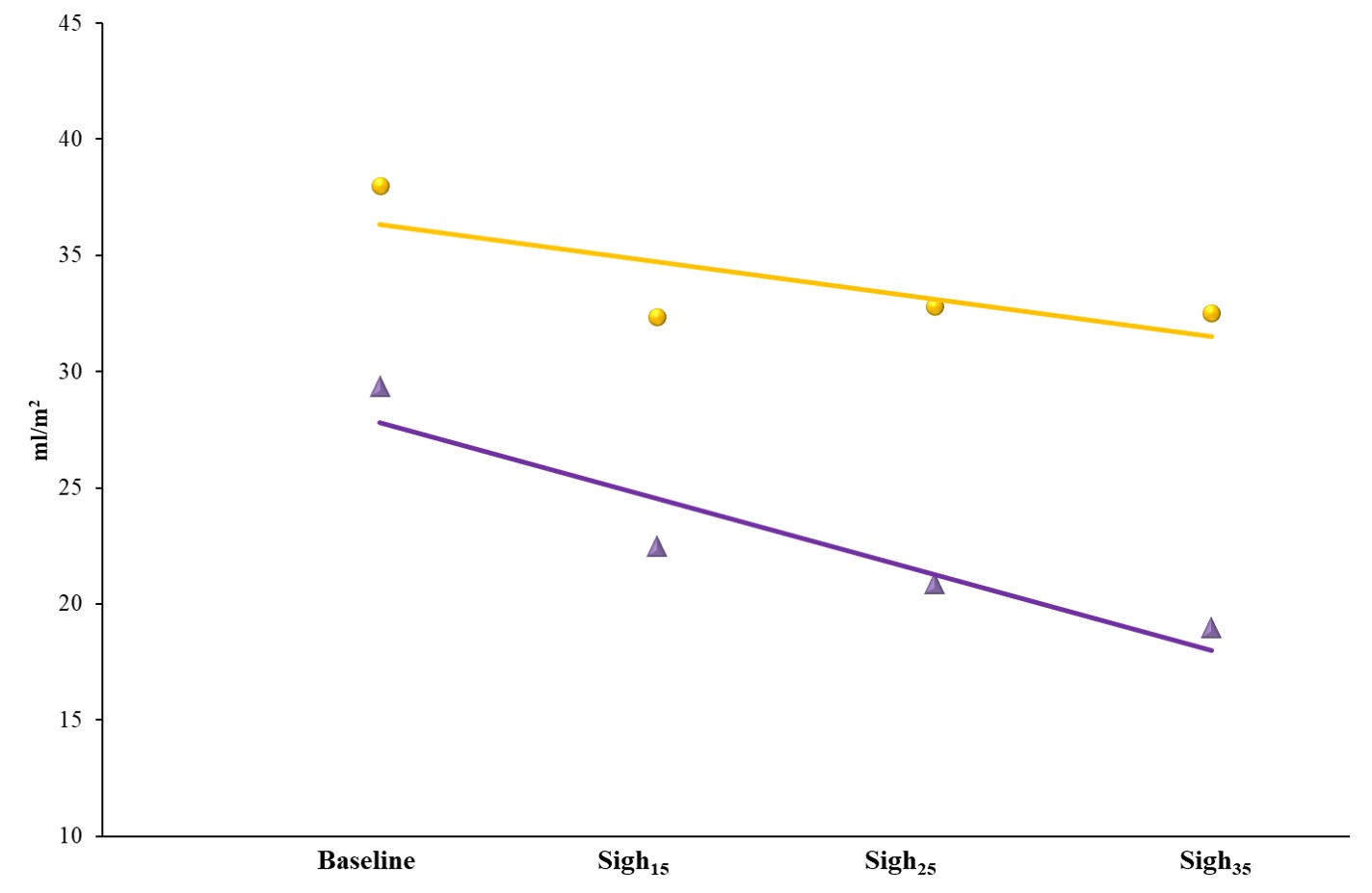

Supplement: Supplementary file 1 — Figures S1 and S2. Slope calculation of pulse pressure (PP) and stroke volume index (SVI) in responders and non-responders. Red triangles and green circles represent the mean values of PP the two populations (responders and non-responders, respectively) at each step of the protocol. Purple triangles and yellow circles represent the mean values of SVI the two populations (responders and non-responders, respectively) at each step of the protocol. For slope computations, please refer to Table 3. (ZIP 57 kb) [file 13054_2018_2294_MOESM1_ESM.zip › Suppl_fig_2_SVI.jpg]
