# Supplementary material for: Sigh maneuver to enhance assessment of fluid responsiveness during pressure support ventilation
Source: Crit Care. 2019 Jan 28;23:31. doi: 10.1186/s13054-018-2294-4 (PMC6350369; doi:10.1186/s13054-018-2294-4)
Supplement: Supplementary file 2 — Table S1. Hemodynamic baseline values at each step of the protocol. (DOCX 15 kb) [file 13054_2018_2294_MOESM2_ESM.docx]

Table S1. Hemodynamic baseline values at each step of the protocol.

| **Hemodynamic variables** | **pre SIGH_15_** | **pre SIGH_25_** | **pre SIGH_35_** |
| --- | --- | --- | --- |
| **CI (L/min/ m^2^)** |  |  |  |
| Responders | 2.0 [1.7 – 2.4] | 2.0 [1.7 – 2.2] | 2.1 [1.9 - 2.4] |
| Non-responders | 2.7 [2.3 - 3.2] | 2.7 [2.3 - 3.2] | 2.7 [2.4 - 3.2] |
| **SVI (mL/ m^2^)** |  |  |  |
| Responders | 23 [20 - 29] | 22 [20 - 26] | 24 [21 – 29] |
| Non-responders | 38 [31 - 51] | 38 [31 - 48] | 40 [31 - 49] |
| **MAP (mmHg)** |  |  |  |
| Responders | 76 [71 - 81] | 75 [70 - 81] | 74 [69 - 79] |
| Non-responders | 76 [66 – 88] | 76 [67 - 89] | 78 [68 - 88] |
| **SAP (mmHg)** |  |  |  |
| Responders | 113 [108 - 117] | 112 [107 - 115] | 108 [104 - 116] |
| Non-responders | 116 [106 – 142] | 118 [107 - 142] | 122 [108 - 144] |
| **HR (beats/min)** |  |  |  |
| Responders | 89 [84 - 92] | 90 [85 - 92] | 91 [84 – 93] |
| Non-responders | 69 [61 - 84] | 70 [62 - 84] | 70 [61 - 82] |

Data presented as median (25^th^ – 75^th^ IQR). CI, cardiac index; SVI, stroke volume index; MAP, mean arterial pressure; SAP, systolic arterial pressure; HR, heart rate.
